# Sex Differences in Collateral Circulation and Outcome After Mechanical Thrombectomy in Acute Ischemic Stroke

**DOI:** 10.3389/fneur.2022.878759

**Published:** 2022-05-19

**Authors:** Christian Lagebrant, Birgitta Ramgren, Ashkan Hassani Espili, Antonio Marañon, Christine Kremer

**Affiliations:** ^1^Medical Faculty, Lund University, Lund, Sweden; ^2^Department of Diagnostic Radiology, Neuroradiology, Department of Clinical Sciences, Lund University, Lund, Sweden; ^3^Department of Statistics, Lund University, Lund, Sweden; ^4^Neurology Department, Department of Clinical Sciences, Skåne University Hospital Malmö, Lund University, Lund, Sweden

**Keywords:** ischemic stroke, mechanical thrombectomy, collateral flow, outcome, sex differences

## Abstract

**Background:**

Collateral circulation is known to lead to smaller infarct volume and better functional outcome after mechanical thrombectomy (MT), but studies examining sex differences in collateral circulation are scarce. The aim of this study was to investigate if collateral circulation has a different impact on outcome in women and men.

**Methods:**

A single-center retrospective study of 487 patients (230 men and 257 women) treated with MT for acute ischemic stroke in the anterior cerebral circulation. Collateral circulation was assessed on computed tomography angiography images. The outcome was evaluated at 90 days according to the modified Rankin Scale (mRS).

**Results:**

Women were older, median age 76 years (IQR 68-83) vs. 71 years (IQR 63–78). Stroke severity and time to recanalization were comparable. More women had moderate or good collaterals in 58.4 vs. 47.0% for men (*p* = 0.01). Among patients with moderate and good collaterals significantly more men (61%) were functionally independent (mRS 0–2) than women (41.5%) (*p* = < 0.01). This difference remained significant after correcting for age by linear weighting, 60.4 vs. 46.8% (*p* = 0.03).

**Conclusion:**

Women had better collateral flow but showed worse functional outcomes, while good collateral flow led to better outcomes in men, even after correcting for age. Further clinical studies on peri- and post-interventional care, factors affecting recovery after hospital discharge as well as basic research on the neurovascular unit are needed to find modifiable targets to improve clinical outcomes for women.

## Introduction

There is a growing awareness that stroke etiology, incidence, and outcome differ between men and women. Women have a higher lifetime risk of stroke than men due to their longer life expectancy and a higher incidence of stroke at older ages (1–3). There are also differences in stroke risk factors, where women have a higher prevalence of hypertension and atrial fibrillation, whereas cardiovascular disease, large artery atherosclerosis, smoking, and alcohol use are more prevalent among men (2). Furthermore, women have worse functional outcomes compared to men. A large part of these differences could be explained by age, stroke severity, and pre-stroke comorbidity. But even after adjustment for these factors, women have a higher disability and lower quality of life after stroke than men (1–3).

In 2015, randomized controlled trials (RCTs) proved that mechanical thrombectomy (MT) is more effective than IVT in stroke caused by occlusion of the anterior cerebral circulation if administered within 6 h after stroke onset (4, 5). Since the publication of these studies, the utilization of MT has increased widely (6). Recent studies also demonstrated that MT is effective up to 24 h after onset of stroke in selected patients with radiological imaging showing ischemic but not yet infarcted brain tissue (7, 8). There has been some uncertainty about whether the outcome after MT is affected by the sex of the patient. One *post-hoc* analysis of data from a large RCT found that women were less likely to benefit from MT and that women had higher mortality and more adverse events than men (9). Another prospective cohort study reported that women were less likely to be functionally independent 90 days after MT compared to men (10). However, several other studies, including one large recently published meta-analysis of seven RCTs, did not confirm these findings and concludes that sex does not influence clinical outcome after MT (11–13).

It has been shown that collateral circulation influences outcome after MT, where a good collateral status leads to smaller infarct volume and better functional outcome (14, 15). The anatomy of these alternative pathways differs greatly between individuals. Experimental studies in rodents have found no structural or functional differences in leptomeningeal collateral circulation between sexes, nor any anatomical differences in the circle of Willis between men and women (16, 17). In animal studies, the amount of cerebral collaterals is determined by multiple factors, genetic as well as environmental. Age and comorbidities such as hypertension, endothelial dysfunction, diabetes, and obesity are known to cause loss of collateral number, a decrease in collateral vessel diameter, and impaired autoregulation of cerebral vasculature (17, 18). Just a few studies include collateral circulation as a prognostic parameter for outcomes like the meta-analysis of seven RCTs by Chalos et al. (12). In this study, they found that women included in the randomized MT trials had better collateral status than men. Interestingly, this did not improve the functional outcomes for these women.

This study aims to evaluate collateral circulation in patients receiving MT for stroke in the anterior cerebral circulation and to investigate if the collateral status has a different impact in women and men.

## Method

This study is a retrospective evaluation of patients receiving MT between 1 January 2014 and 31 December 2018. The medical imaging program Picture Archiving and Communication System (PACS) was used to identify all referrals for MT within this period. Only patients with stroke in the anterior cerebral circulation were included in the study. Cases, where no attempt of MT was made, were excluded. Patients who suffered a second stroke and had a second MT during the 90-day follow-up were also excluded from this study. Cases with missing or technically failed computed tomography angiography (CTA) were also excluded.

### Clinical Data

Clinical baseline data included: age, sex, initial National Institute of Health Stroke Scale (NIHSS) score, time of stroke onset, time from onset to recanalization—if the time of onset was unknown, a time when the patient was last seen well was used—and neurological impairment at admission according to NIHSS was evaluated (19). Clinical data were evaluated by assessing the local database, and time from onset and time to recanalization were reviewed in PACS together with the recanalization outcome.

### Radiological Data

Computed tomography angiography was performed on multidetector-CT scanners from different companies in place at the 13 referral hospitals within the catchment area, and a standard CTA protocol was used with bolus-tracking software to acquire images at the peak contrast arrival. Collateral circulation was assessed with axial view CTA imaging. In one case, the axial view was missing and the coronal view was used instead. The amount of intra-arterial contrast in the area distal to the occlusion was evaluated. The contralateral side of the brain was used as a baseline for comparison. Collateral circulation was graded and given a score of 0–3 with a method described by Tan et al. (20):

0 = No collateral circulation = absent collaterals in the vascular territory supplied by the occluded arterial segment.

1 = Poor collateral circulation = collaterals filling <50% of the vascular territory supplied by the occluded arterial segment.

2 = Moderate collateral circulation = collaterals filling more than 50%, but <100% of the vascular territory supplied by the occluded arterial segment.

3 = Good collateral circulation = collaterals filling 100% of the vascular territory supplied by the occluded arterial segment.

Computed tomography angiography imaging was reviewed using PACS on computers and monitors used professionally in clinical practice by radiologists in SUS Lund. Collateral circulation was assessed by the authors (CL and BR) in all included patients. All inconclusive cases were reviewed together with a senior neuroradiologist (BR) to ensure high-quality assessments throughout the material (Supplementary Figure 1).

### Outcome Data

One measure of outcome analyzed in this study was thrombolysis in cerebral infarction (TICI) after recanalization with MT. TICI is a radiological scale describing perfusion past an occlusion on angiography ranging from 0 to 3. TICI 2B, 2C, and 3 are defined as successful recanalization (21, 22). TICI-scores were collected from post-interventional charts in PACS.

The main outcome in this study was patient functional status and independence 90 days after stroke according to the modified Rankin Scale (mRS) (23).

A 90-day mRS-score was obtained by interpreting data from Riksstroke, a Swedish national quality registry for stroke care. All hospitals treating acute stroke in Sweden are submitting data to this register in a standardized manner (6). Information on activities of daily living (ADL), such as personal hygiene, dressing, and mobility, is recorded in a 90-day follow-up. By using syntax, we converted the ADL-data into the mRS.

For patients with no 90-day follow-up recorded in the Riksstroke registry, mRS-scores were obtained, if available, by interpreting patient medical records.

### Statistical Analysis

Baseline demographics and clinical characteristics were expressed as median values with an inter-quartile range (IQR). The Mann-Whitney *U*-test was used to compare average ranks for clinical baseline data between men and women.

The chi-square test was used to test for dependence between sex and distribution of collateral status and TICI-score. The chi-square test was also used to test for sex differences in the mRS score 90 days after MT for different degrees of collateral circulation. Analysis of collateral circulation was made for each separate group of collateral circulation as well as for dichotomized groups of none and poor vs. moderate and good collateral circulation. To correct for age, we applied linear weighting and matching. Essentially the algorithm tries to find the optimal set of weights with minimal variance so that the resulting weighted means and standard deviations for age, as well as the percentage distributions for the age quartile groups, are similar in both gender categories. The weights are obtained using the GfK linear weighting and matching program.

The SPSS Statistics for Windows version 27 (IBM Corporation, Armonk, NY, USA) was used for all statistical analyses. *P* < 0.05 were considered to be significant.

This study was approved by the Research Ethics Committee in Lund with registration numbers 2013/466 and 2018/53.

## Results

A total of 487 of 751 referrals for MT met inclusion criteria, 230 (47%) men and 257 (53%) women. (PRISMA flow chart, Supplementary Figure 2). Clinical characteristics are summarized in Table 1.

**Table 1:** Clinical characteristics by sex.

**Characteristics**	**Men, median (*n* = 230)**	**IQR**	**Women, median (*n* = 257)**	**IQR**	***P*-value^a^**
Age (years)	71 (*n* = 230)	63–78	76 (*n* = 257)	68–83	<0.01
NIHSS at admission (points)^b^,^c^	15 (*n* = 217)	11–19	15 (*n* = 246)	10–18	0.41
Time from onset to recanalization (h:min)^d^	4:39 (*n* = 181)	3:40–5:56	4:41 (*n* = 198)	3:39–5:49	0.76

a *Mann Whitney U-test for differences in median value between men and women*.

b *NIHSS, National Institutes of Health Stroke Scale*.

c *Data missing for 24 patients, 11 women and 13 men*.

d *Data missing for 108 patients, 59 women and 49 men*.

Women in our study were significantly older with a median age of 76 years (IQR 68–83) vs. 71 years (IQR 63–78) for men (*p* = < 0.01). The severity of stroke did not differ between the sexes. Median NIHSS before MT was 15 points for both men (IQR 11–19) and women (IQR 10–18). The time from onset of stroke to recanalization was similar between the groups.

Women had better collateral circulation status (Table 2). When dichotomized into groups of no or poor collateral circulation and moderate or good collateral circulation, more women had moderate or good collateral 58.4 vs. 47.0% for men (*p* = 0.01).

**Table 2:** Collateral circulation in patients receiving mechanical thrombectomy.

**Collateral status**	**Men (*n* = 230)**	**Women (*n* = 257)**	***P*-value**
No collateral circulation	28 (12.2%)	24 (9.3%)	
Poor collateral circulation	94 (40.9%)	83 (32.3%)	
Moderate collateral circulation	43 (18.7%)	64 (24.9%)	
Good collateral circulation	65 (28.3%)	86 (33.5%)	0.09^a^
No/poor collateral circulation	122 (53.0%)	107 (41.6%)	
Moderate/good collateral circulation	108 (47.0%)	150 (58.4%)	0.01^b^

a *Chi 2-test for sex difference in distribution among collateral circulation groups*.

b *Chi-2 test for sex difference after dichotomization of collateral circulation groups*.

### Outcome Measures

There was no sex difference in radiological outcome after MT according to TICI-scale. Seventy-seven percent of women had successful recanalization, with a TICI-score of 2B, 2C, or 3, compared to 79.0% for men.

More men were functionally independent 90 days after MT, with mRS 0–2, 45.2% compared to 35.0% for women. A 90-day mortality, mRS 6, was higher for women, 24.0 vs. 18.8%. The proportion of disabled patients, mRS 3–5, was higher for women, 41.0 vs. 36.0% for men (Table 3).

**Table 3:** Functional status 90 days after mechanical thrombectomy.

**Modified rankin scale^a^**	**Men (*n* = 208)**	**Women (*n* = 246)**	***P*-value^b^**
0–2	94 (45.2%)	86 (35.0%)	
3	22 (10.6%)	36 (14.6%)	
4	34 (16.3%)	34 (13.8%)	
5	19 (9.1%)	31 (12.6 %)	
Dead	39 (18.8%)	59 (24.0%)	0.11

a *Data missing for 33 patients, 11 women and 22 men, who had no 90-day follow up recorded in Riksstroke*.

b *Chi 2-test for sex differences in distribution among groups of mRS90*.

**Figure 1:**
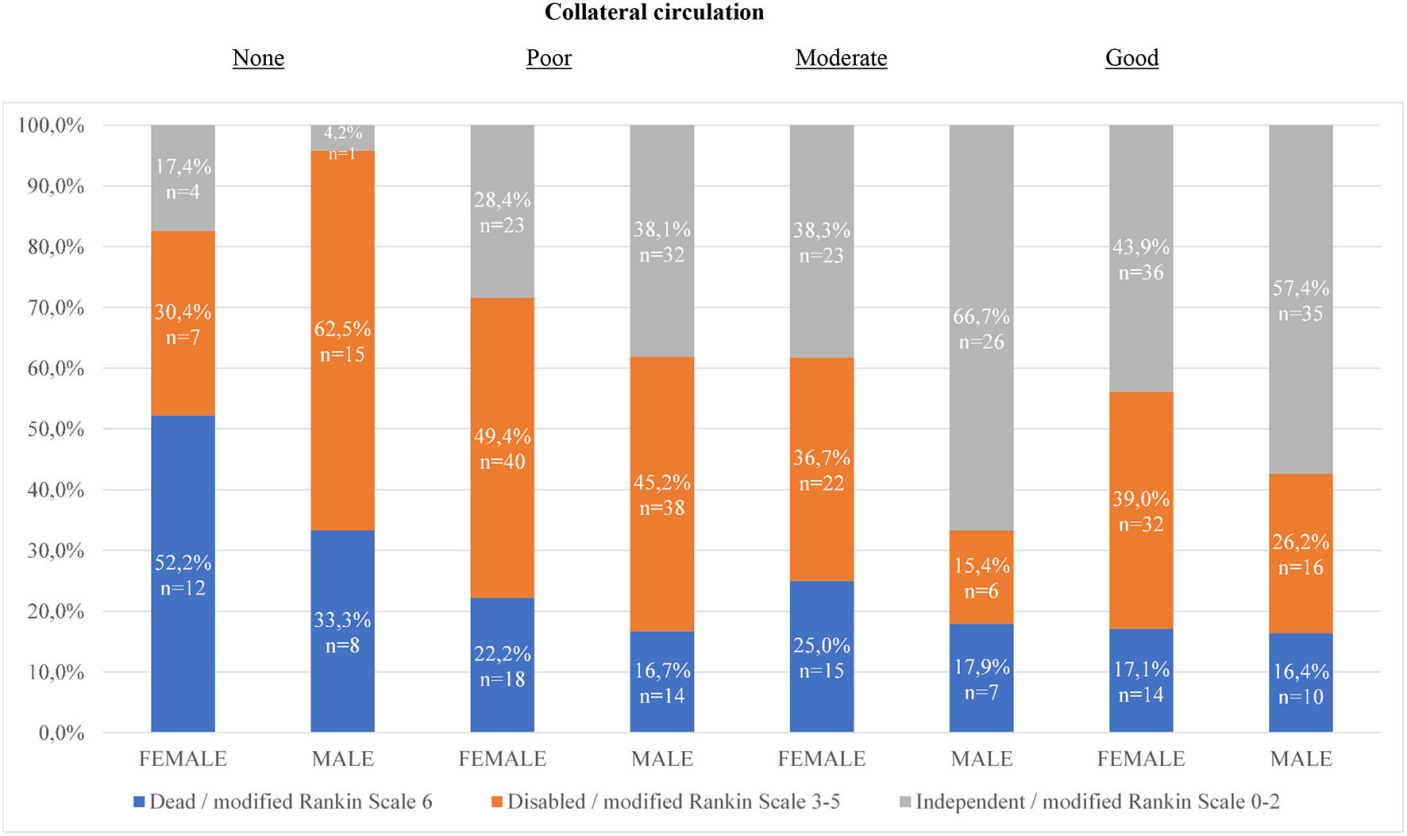
Distribution of functional outcomes 90 days after mechanical thrombectomy for different degrees of collateral circulation. Chi 2-test for sex differences in proportion of functionally independent outcome: 1. (*p* = 0.14) 2. (*p* = 0.19) 3. (*p* = 0.01) 4. (*p* = 0.11) For dichotomization moderate and good collaterals together (*p* = < 0.01). For dichotomization none and poor collaterals together (*p* = 0.46).

In Figure 1, the distribution of functional outcomes 90 days after MT is presented for different degrees of collateral circulation. In the groups with poor, moderate, and good collateral circulation, men were functionally independent to a higher extent than women. The differences in outcome were particularly evident in the groups with moderate and good collateral circulation. When these two groups were combined into one, 61.0% of men were functionally independent compared to women 41.5% (*p* = < 0.01). In the group of patients with no collateral circulation, women were independent to a higher extent than men. This difference was however not significant.

Since the age distributions were significantly different for the gender groups of patients, we investigated if controlling for age in both gender groups can affect the percentages of functional independence among patients with moderate and good collateral circulation after the application of linear weighting and matching on the 487 patients. Applying the derived linear weights, the results for the percentages of functionally independent outcomes 90 days after MT were as follows: 60.4% of the male patients were functionally independent 90 days after MT, while the corresponding percentage for women was estimated at 46.8%. The difference was found to be statistically significant, following the chi-square test, with *p* = 0.034.

## Discussion

In this single-center study, women treated with MT had better collateral status than men, but with worse outcomes. These differences remained significant even after correcting for age. Our results are comparable with the results from the recently published meta-analysis by Chalos et al. (12). This is the largest study on outcome after MT to this date and one of few studies specifying collateral status in their baseline characteristics. Furthermore, using the grading system by Tan et al. (20), they reported that more women had moderate or good collateral circulation grades, 88% of women compared to 80% of men (*n* = 1,290, *p* = < 0.01). The proportion of moderate or good collateral circulation in this meta-analysis is higher for both men and women than in our study (12).

Women showed even better collaterals in the Defuse 3 cohort, a cohort selected by neuroimaging for thrombectomy presenting between 6 and 16 h after stroke (24).

The reasons for these observed disparities in collateral flow between men and women receiving MT are not known. It is interesting that women in our study had better collateral status even though they were 6 years older by median age than the included men. However, it is known that smoking, alcohol use, large vessel atherosclerosis, and peripheral vascular disease are more prevalent in men with stroke than in women with stroke (2). These factors lead to early vessel aging and impaired autoregulation and could potentially explain men's lower collateral scores. Several agents or mechanisms such as steroid- and sex-hormones, sex chromosomes, differences in cell death, differences in immune pathways, epigenetic regulation, and sex-specific microRNAs have all been proposed to cause sex differences in stroke (17, 18). However, the regulation of cerebral vasculature is still poorly understood, and much effort is needed to fully comprehend these mechanisms. Further research to clarify the question of potential sex differences in collateral circulation is of utmost importance, as pharmacological regulation of collateral vessels has been proposed as a possible therapeutic approach in acute stroke treatment (25).

Rates of successful recanalization were similar between men and women in our study. This is in line with the results from several other investigations of outcomes after MT, where no sex difference in TICI-score after MT was found (11–13). Altogether this suggests that it is not the intervention itself that causes disparities in outcomes and that women should not be withheld from MT treatment on the sole basis of their sex.

There might also be aspects of peri- and post-interventional care explaining sex differences. Blood pressure during and after stroke is known to affect the outcome. Both hypotension and hypertension are prognostic factors for poor outcomes (26). Current guidelines recommend permissive hypertension, that is allowing blood pressure up to 180/105 mm Hg in the first 24 h after IVT or MT to ensure increased perfusion to the affected area (27). However, specific evidence on blood pressure after MT is lacking. One study has found that high peak values of systolic blood pressure during the first 24 h after MT correlated with the worse functional outcomes as well as with higher rates of hemorrhagic complications (28). Another study found that variabilities in systolic blood pressure after MT are associated with worse clinical outcomes (29). In some women, there could also be a risk of hypotension due to heart failure induced by atrial fibrillation, a disease more common among women with stroke (2). It is therefore possible that difficulties in regulating peri- and post-interventional blood pressure in these women could be a factor contributing to sex difference in the outcome.

The differences seen in our study are likely multifactorial, where age and pre-stroke comorbidity might offer some causative explanations. But it is also possible that the worse functional outcome seen in women is a result of unmeasured contributors affecting recovery post-stroke and after hospital discharge. Except for the already discussed factors, some researchers have proposed musculoskeletal comorbidities such as arthritis and osteoporosis (10). These diseases are known to be more common in women than in men and could influence the mRS 90 days after stroke by increasing complications as well as limiting the possibilities for rehabilitation and mobilization.

Our study has several limitations. The sample size of included patients is relatively low, and the study could be underpowered to properly detect differences between men and women.

Another weakness is that we had limited information on the pre-morbid status or cognitive decline of patients. Some differences in outcomes might have been influenced by factors not captured in the analysis, even though this might not explain the differences in recruitment of collateral flow and affect the 3-month outcome.

Assessment of collateral circulation was made by the CL after training and, in non-conclusive cases, with the assistance of a senior neuroradiologist. The images were not blinded to clinical data or outcome. The CTA images used to assess collateral circulation were of varying quality. The purpose of CT and CTA exams performed in an acute stroke care setting is primarily to find hemorrhage and site of occlusion and not to assess collateral circulation. Thus, CTA imaging with poorly timed contrast delivery—for example, due to technical problems with the CT machine, due to low cardiac output in a patient with chronic heart failure, or due to asymptomatic stenosis on the extracranial part of the carotid artery—could be enough to visualize the occluded vessel but at the same time not enough to properly display collateral circulation. We excluded all such CTA images of low technical quality from the analysis. But it might still be possible that temporal delay in the filling of collateral vessels could have caused inaccurate visualization of collateral circulation in some cases.

## Conclusion

In summary, women in our study had better collateral circulation but worse functional outcomes.

No collateral circulation led to a high proportion of poor outcomes for both men and women. Women do not have the same beneficial effect of an increasing degree of collateral circulation as men do. Among patients with moderate and good collateral circulation, a significantly higher proportion of men reached functional independence.

## Data Availability Statement

The original contributions presented in the study are included in the article/Supplementary Materials, further inquiries can be directed to the corresponding author.

## Ethics Statement

The studies involving human participants were reviewed and approved by Research Ethics Committee Lund University 2013/466 and 2018/53. Written informed consent for participation was not required for this study in accordance with the national legislation and the institutional requirements.

## Author Contributions

CK and BR contributed to the conception of the study. AH collected and analysed the data. CL and BR reviewed the radiological data. CL wrote the first draft. AM performed the statistical analysis. All authors contributed to the revision of the manuscript.

## Conflict of Interest

The authors declare that the research was conducted in the absence of any commercial or financial relationships that could be construed as a potential conflict of interest.

## Publisher's Note

All claims expressed in this article are solely those of the authors and do not necessarily represent those of their affiliated organizations, or those of the publisher, the editors and the reviewers. Any product that may be evaluated in this article, or claim that may be made by its manufacturer, is not guaranteed or endorsed by the publisher.
