# Supplementary material for: Sex Differences in Collateral Circulation and Outcome After Mechanical Thrombectomy in Acute Ischemic Stroke
Source: Front Neurol. 2022 May 19;13:878759. doi: 10.3389/fneur.2022.878759 (PMC9160377; doi:10.3389/fneur.2022.878759)
Supplement: Supplementary file 1 [file Data_Sheet_1.PDF]

Fig 1. Flow chart presenting patients included and excluded in the analysis

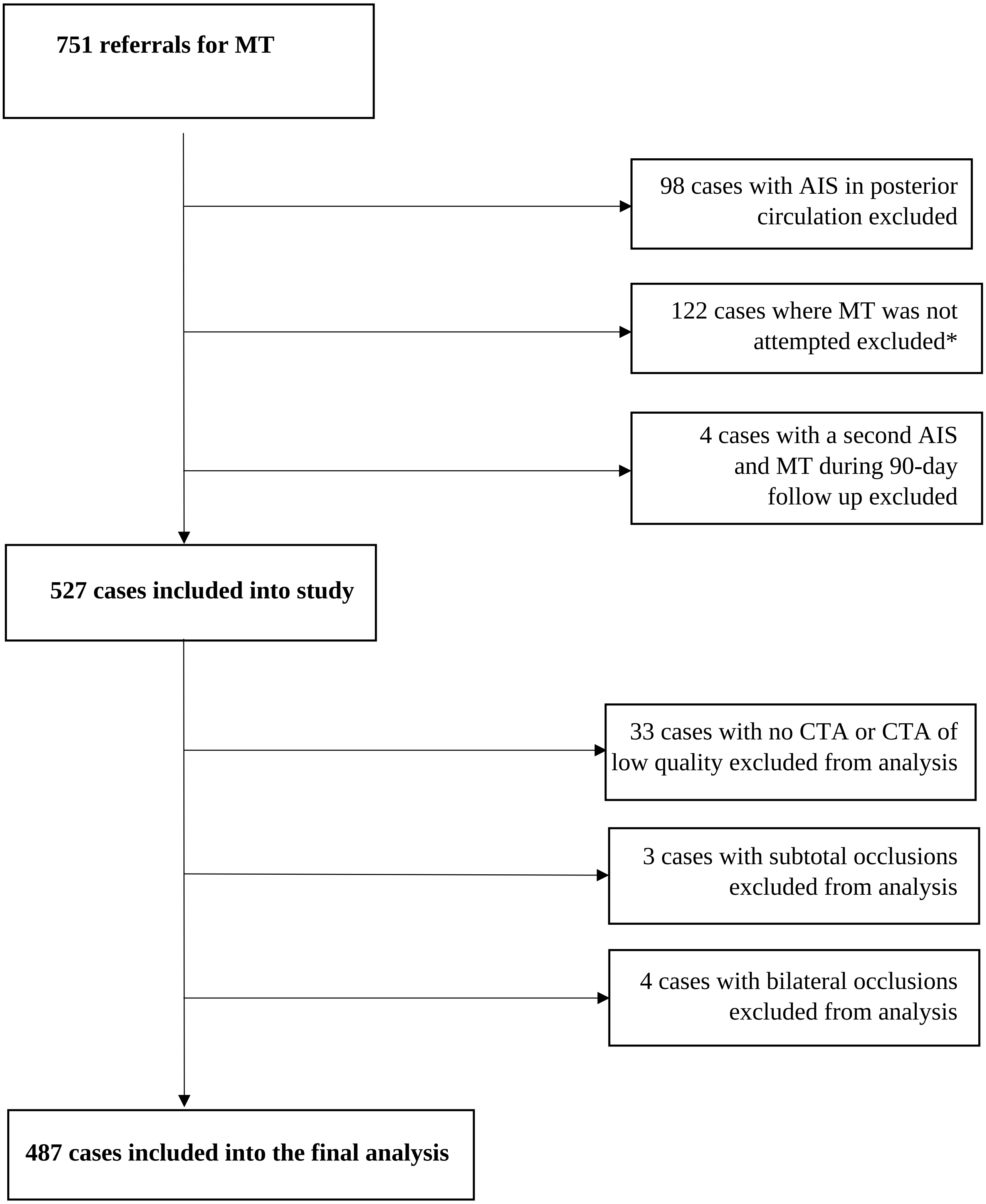

MT mechanical thrombectomy, AIS acute ischemic stroke * i.e. due to spontaneous recanalization, recanalization by IVT, failure to access femoral artery, failure to catheterize intracranial arteries due to anatomical difficulties, extracranial stenting without intracranial thrombectomy.

Fig. 2 CT- angiography images of a patient with good collateral flow (Grade 3) (A) and no collateral flow (Grade 0) (B) according to TAN et al.

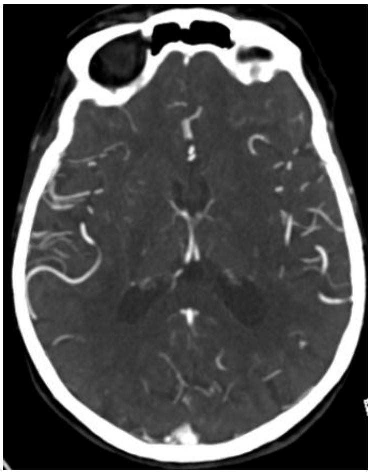

3 = Good collateral circulation = collaterals filling 100 % of the vascular territory supplied by the occluded arterial segment

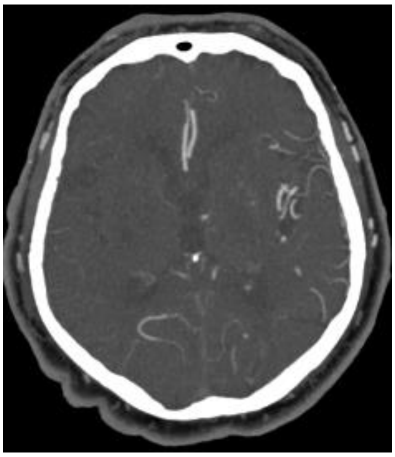

0 = No collateral circulation = absent collaterals in the vascular territory supplied by the occluded arterial segment
